# Age-Dependent Remodeling of Parvalbumin Interneuron-Associated Networks Alters Corticospinal Output in the Motor Cortex

**DOI:** 10.3390/cimb48100978

**Published:** 2026-09-23

**Authors:** Xiaofei Wei, Istvan Mody, Yixin Wu, Aryan Gajjar, Gurnoor Singh, Vignesh Neerathalingam, Adeline Sun, Ruyi Huang, Sandra M. Holley, Daniel C. Lu

**Affiliations:** 1 Department of Neurosurgery, David Geffen School of Medicine, University of California Los Angeles (UCLA), Los Angeles, CA 90095, USA; wuyixin0909@gmail.com (Y.W.); agajjar1@g.ucla.edu (A.G.); noorsingh1649@gmail.com (G.S.); vigneer21@gmail.com (V.N.); ruyihuang@mednet.ucla.edu (R.H.); sholley@mednet.ucla.edu (S.M.H.); 2 Department of Neurology, David Geffen School of Medicine, University of California Los Angeles (UCLA), Los Angeles, CA 90095, USA; mody@ucla.edu (I.M.); adelinesun@gmail.com (A.S.); 3 Department of Anatomy and Neurobiology, University of California-Irvine, Irvine, CA 92617, USA

**Keywords:** parvalbumin interneuron, primary motor cortex, corticospinal tract, aging, optogenetics, electromyography

## Abstract

The primary motor cortex (M1) controls voluntary movements through coordinated interactions between excitatory pyramidal neurons and inhibitory parvalbumin-expressing interneurons (PV-INs). Although PV-INs are critical to regulating motor output and motor coordination, their role in age-related motor decline remains unclear. Here, we investigated how aging alters PV-IN-associated regulation of corticospinal tract (CST) output in mice. Aged mice (14–24 months) exhibited selective motor impairments, including deficits in balance and hindlimb-supported performance, while general locomotor activity remained largely preserved. We combined optogenetics, ex vivo electrophysiology, retrograde tracing, and in vivo electromyographic (EMG) recordings. In aged M1 slices, optogenetic stimulation of the PV-IN-targeted network produced small extracellular-like voltage deflections recorded with a loose-apposed, non-sealed pipette configuration. These responses persisted during GABA_A_ receptor blockade but were abolished by DNQX and APV, indicating engagement of an ionotropic glutamate receptor-dependent network mechanism. Whole-cell recordings obtained in separate experiments demonstrated that conventional inhibitory PV-IN-to-pyramidal transmission remained detectable during repetitive stimulation. In vivo, PV-IN activation increased M1-evoked hindlimb EMG responses in aged mice but had little effect in young animals. Together, these findings identify an age-dependent reorganization of PV-IN-associated motor-cortical network function that alters the recruitment of glutamatergic mechanisms and corticospinal motor output. The present data do not establish the precise synaptic topology of this network response or whether the reorganization is compensatory or maladaptive.

## 1. Introduction

The primary motor cortex (M1) is a central hub for motor planning, execution, and adaptation [1]. Its function relies on coordinated interactions between excitatory pyramidal neurons and diverse populations of inhibitory interneurons that regulate neuronal excitability, temporal precision, and network synchronization [2]. Among cortical interneurons, parvalbumin-expressing interneurons (PV-INs) represent the largest subtype (~40% of inhibitory neurons) in the cortex [3,4]. PV-INs form dense synaptic connections onto the soma and proximal dendrites of pyramidal neurons, positioning them as powerful regulators of cortical output [5]; they also interact extensively with other interneuron populations. Through their fast-spiking properties and extensive connectivity [6], PV-INs synchronize pyramidal neuron activity and contribute to the precise timing required for motor coordination and skilled movement.

Within M1, layer V pyramidal neurons provide the major cortical origin of the corticospinal tract (CST), the principal descending pathway linking motor cortex to spinal motor circuits [7]. Their axons descend from M1 through the brainstem into the spinal cord, where they terminate within the spinal gray matter and form synaptic connections predominantly with spinal interneuron and premotor circuits that, in turn, regulate motoneuron activity [8]. Thus, cortical layer V pyramidal neurons provide a direct anatomical route through which local M1 microcircuits can influence limb motor output. PV-INs form dense peri-somatic and proximal-dendritic synapses onto layer V pyramidal neurons and are therefore strategically positioned to regulate the timing and gain of corticospinal output [5,9]. Beyond suppressing excitation, PV-INs shape cortical information processing by regulating network oscillations, synchronizing neuronal ensembles, and improving the fidelity of cortical output signals. Disruption of PV-IN function has been implicated in neurological and neurodevelopmental disorders characterized by motor dysfunction, including autism spectrum disorders, epilepsy, and stroke [10]; however, their role in age-related motor decline remains poorly understood.

Aging is associated with progressive impairments in motor performance, including deficits in balance, coordination, gait stability, and movement precision [11]. These functional declines are associated with alterations in motor-cortical activity and CST function [12]. Increasing evidence suggests that aging is also accompanied by substantial reorganization of inhibitory circuits, including changes in interneuron connectivity, synaptic transmission, and excitatory–inhibitory (E/I) balance [13]. Such alterations may profoundly influence how motor commands are processed and transmitted through cortical networks. Notably, studies of motor recovery after stroke have demonstrated that targeted manipulation of inhibitory interneuron populations can restore motor function by re-establishing appropriate E/I balance and network dynamics [14,15]. These findings raise the possibility that adaptive changes within inhibitory circuits may similarly influence motor function during normal aging. Despite the recognized importance of PV-INs in motor-cortical processing, little is known about how aging affects PV-IN-mediated regulation of CST output. In particular, it remains unclear whether age-related motor deficits are associated with alterations in PV-IN connectivity, synaptic signaling, or network function within M1. Addressing these questions is essential to understanding how cortical microcircuits adapt to age-related challenges and whether inhibitory interneurons contribute to compensatory mechanisms that preserve motor function.

In the present study, we combined behavioral analysis, optogenetics, ex vivo electrophysiology, retrograde tracing, and in vivo electromyographic recordings to investigate how aging alters PV-IN-associated regulation of CST output. We show that aging is associated with selective deficits in balance and hindlimb-supported motor performance together with an age-dependent change in the cortical network response engaged by PV-IN optogenetic stimulation. Conventional inhibitory transmission from PV-INs to pyramidal neurons remained detectable, whereas optogenetic stimulation of the PV-IN-targeted network in aged M1 additionally engaged an ionotropic glutamate receptor-dependent response and increased M1-evoked hindlimb motor output. We therefore interpret these findings as evidence of age-dependent reorganization of PV-IN-associated motor-cortical network function while recognizing that the underlying synaptic topology and its adaptive versus maladaptive significance remain to be determined.

## 2. Materials and Methods

### 2.1. Animals

This study used a hybrid mouse line, PV-cre/Ai27 (Jackson Strain #017320/#012567) which expresses channelrhodopsin-2/tdTomato fusion protein on PV-INs, or PV-cre/Ai39 (Jackson Strain #017320/#014539), which expresses Halorhodopsin/EYFP fusion protein on PV-INs. Experimental mice were divided into 2 groups: aged (14–24 months old) and young adult (3–6 months old) groups. Animals were housed with ad libitum access to food and water under the care of the UCLA Division of Laboratory Animal Medicine (DLAM). Mice were maintained on a reversed light/dark cycle of 12 h (light started at 9 p.m.). All procedures were performed in accordance with the protocol approved by the Institutional Animal Care and Use Committee (IACUC) and guidelines of the National Institutes of Health.

### 2.2. Behavioral Tests

All behavioral tests were performed during the animal’s dark cycle (9 a.m.–9 p.m.). For all behavioral tests, mice were allowed a habituation period in the testing room in their home cages for at least 1 h. The test animal had another break for at least another 1 h between different tests if more than one test was performed on the same day. The surfaces for all the behavioral tests were carefully sanitized using 75% ethanol between animals to remove any odor and odorant cues.

Grid walking. A custom-designed setup was built to obtain 3D information using only one camera [16]. Two side mirrors were used along the shorter and longer sides of a transparent cage with wire mesh at the bottom. A top-mounted camera captured the movement of the animal on the wire mesh located in the central part of the image, while the two mirrored side-view images were projected on the two sides of the video image. The central image provided information about the animal’s speed, location and orientation; the mirrored images were used to detect the movement of a specific body part through the grid, i.e., depth, duration and 1-dimensional side-projected location. A custom-developed program in Igor Pro 6.32 (Wavemetrics, Lake Oswego, OR, USA) was used to analyze the video recordings. The combination of the three image segments yielded the 3D data necessary to determine the exact body part (snout/head, tail or limbs) protruding through the grid, hereafter referred to as a ‘fault’. Multiple parameters could thus be derived based on the 3D information: total travel distance; average speed; time-instantaneous speed; fault number; fault–speed relation; animal location trajectory; center–peripheral location preferences; fault locations on the grid; normalized relative fault location to the body part; fault duration–depth matrices for each limb; distribution of the fault depth and fault durations for each limb, head and tail; grouping the faults based on their depth and duration; animal rotations; etc.

Balance beam. The apparatus for the beam-walking test consisted of a round horizontal bar 100 cm long and 20 mm in diameter and was elevated 50 cm in a second container; a cushion was placed under the beam to protect the mice in case of falling. Two different types of beams with different surface materials were used: smooth surface and rough surface. A stop was fixed to each end of the beam (see picture in Figure 1B). A top-mounted camera captured the movement of the animals on the beam, and a custom-developed program in Igor Pro 6.32 (Wavemetrics, Lake Oswego, OR, USA) was used to do the post-analysis of the video recordings. For the test trial, each animal was placed at the center of the beam and was allowed to freely explore for 1 min. Animals that successfully remained on the beam after the exploration period were recorded for up to 2 min. Trials were terminated immediately if the animal fell from the beam during the recording period. Behavioral parameters, including normalized total travel distance on the beam, average locomotor speed, and the center-to-periphery occupancy ratio, were quantified for analysis.

Rope pulling. The test method was modified from Khademullah’s study [17]. The testing apparatus was a transparent polyethylene cylinder (15 cm in diameter) with a thin stick crossing at the top (35 cm in height). One end of the rope (3 mm in diameter) was attached and turned over the stick and loosely fixed at the center. To eliminate potential distraction, 3 walls (rear and sides, leaving the top uncovered) were assembled out of white foam board around the apparatus (Figure 1C). Mice were allowed a 5 min habituation period in the cylinder with the string placed inside prior to recording. String movement was towards the floor of the apparatus if the animal performed the pulling behavior. Before recording, the rope pulled during the habituation period was reset to the starting point, and the recordings were made with a camera placed on one side of the cylinder (15 cm away from recording table) for 2 min. After testing, mice were returned to their home cages. To assess forelimb function, the length of the string pulled during the 2 min recording was documented in cm. Time was recorded for the period during which the mouse was gripping the string with either one or both paws and engaged in a pulling behavior. To assess hindlimb function, time was recorded for the period during which the mouse was gripping the string with either one or both paws while in standing position on its hind paws. Time spent pulling the string while in a seated or crouched position was recorded as time spent pulling but not time spent standing on hindlimbs.

### 2.3. Motor Output Recordings and Stimulation

In vivo recordings were performed on anesthetized animals. Mice were initially anesthetized with isoflurane, followed by intraperitoneal administration of urethane (1.5 g/kg; U2500, Sigma-Aldrich, St. Louis, MO, USA) for sustained anesthesia [18]. The body temperature was maintained at 37.0 ± 0.5 °C using a water-circular heating pad throughout the procedure.

Electrode placement and stimulation protocols: A cranial window (2 mm × 2 mm) was opened over the left primary motor cortex (center: AP: 0 mm; ML: 1.5 mm) to accommodate recording electrodes and optical fibers. Opto-electrode design and stimulation: A custom-built opto-electrode was used for combining electrical and optical stimulation. The electrode consisted of two bare-tipped tungsten wires (0.005 inch in diameter) paired with 10 mm long optical fibers (100 μm core diameter; Thorlabs Newton, NJ, USA; cat # CFMLC21L10). The opto-electrode was positioned at a depth of 400–500 μm to target layer V pyramidal neurons in the hindlimb representation area of M1 (AP: 0.55 ± 1.04 mm; ML: 1.47 ± 0.32 mm) [19]. Electrical stimulation: A 200 Hz, 500 ms train of biphasic pulses (200 μs duration) was delivered to evoke CST-mediated muscle activity. Stimulation intensity (0.2–0.9 mA) was adjusted to elicit observable right hindlimb withdrawal or twitch. If no movement was detected, the electrode was repositioned iteratively until a response was observed. Optical stimulation: PV-INs were selectively activated using 475 nm light (20 Hz, 5 ms pulses) delivered through the optical fiber. Electromyography (EMG) recording: EMG signals were recorded from the contralateral tibialis anterior (TA) muscle using two FPA-coated silver wires (A-M Systems, Carlsborg, WA, USA, Cat #: 786000) (0.125 mm in diameter, bared 2 mm at the tips) inserted via a 26-gauge needle guide. Wires were bent at the tips to secure placement. The stimulation protocol consisted of three sequential phases designed to assess both baseline and PV-IN-modulated motor responses. First, a control period was established by delivering four electrical stimulations to the motor cortex at 30 s intervals to record baseline CST-mediated muscle activity. Next, during the light stimulation period, another set of four electrical stimulations was administered while simultaneously activating PV-INs through optical stimulation, allowing for observation of how inhibitory interneuron modulation influences motor output. Finally, to isolate the effects of PV-IN activation, a single light-only trial was conducted without concurrent electrical stimulation. This sequential approach enabled systematic comparison of motor responses under control, combining modulation, and PV-IN-specific conditions. The recording was made using the AD instrument octal-bio-amplifier (ADInstruments Ltd., Colorado Springs, CO, USA) and digitized at 10 kHz with a Powerlab digital data acquisition device and LabChart 8.1 software (ADInstruments Ltd., Colorado Springs, CO, USA). EMG activity was analyzed offline with Igor Pro 8 (Wavemetrics, Lake Oswego, OR, USA).

### 2.4. Retrograde Virus Injection and Imaging

Retrograde AAV-CAG-GFP (1.6 × 10^12^ vg/mL; two sites, 350 nL/site) was injected into the lumbar spinal cord at the L2-L4 level. Animals were allowed at least 4 weeks for retrograde expression before patch-clamp experiments. To verify cortical labeling, animals were transcardially perfused with PBS followed by 4% paraformaldehyde (pH 7.3). Brains were removed, postfixed for 24 h in 4% paraformaldehyde, cryoprotected in 30% sucrose, embedded in 10% gelatin, and frozen. Tissue was sectioned into 30 µm coronal sections using a cryostat (Leica CM 1800, Leica Biosystems, Deer Park, IL, USA). Images were acquired at 16-bit resolution using a digital microscope (Echo Revolve, Echo, San Diego, CA, USA).

### 2.5. Ex Vivo Slice Preparation and Electrophysiological Recordings

Mice were anesthetized with isoflurane and decapitated in accordance with protocols approved by the UCLA Chancellor’s Animal Research Committee. Horizontal brain slices (350 μm) containing the primary motor cortex were prepared using a Leica VT1000S vibratome (Leica Biosystems, Deer Park, IL, USA) in ice-cold N-methyl-D-glucamine (NMDG)-based HEPES-buffered cutting solution containing (in mM): 135 NMDG, 10 D-glucose, 4 MgCl_2_, 0.5 CaCl_2_, 1 KCl, 1.2 KH_2_PO_4_, 20 HEPES, and 27 sucrose. The solution was continuously bubbled with 100% O_2_ and adjusted to pH 7.4 and 290–300 mOsm/L. Following sectioning, slices were incubated at 32 °C in reduced-sodium artificial cerebrospinal fluid (ACSF) containing (in mM): 85 NaCl, 25 D-glucose, 55 sucrose, 2.5 KCl, 1.25 NaH_2_PO_4_, 0.5 CaCl_2_, 4 MgCl_2_, and 26 NaHCO_3_. The solution was equilibrated with 95% O_2_/5% CO_2_ and maintained at pH 7.3–7.4. After 30 min, slices were transferred to normal ACSF at room temperature containing (in mM): 126 NaCl, 10 D-glucose, 2 MgCl_2_, 2 CaCl_2_, 2.5 KCl, 1.25 NaH_2_PO_4_, 1.5 sodium pyruvate, 1 L-glutamine, and 26 NaHCO_3_ (pH 7.3–7.4 when equilibrated with 95% O_2_/5% CO_2_).

For electrophysiological recordings, slices were transferred to a submerged recording chamber maintained at 34 °C and continuously perfused with ACSF at 5 mL/min. All salts were purchased from Sigma-Aldrich (Sigma-Aldrich, St. Louis, MO, USA). Slices were visualized using infrared differential interference contrast (IR-DIC) microscopy on an upright microscope (Olympus BX-51WI, Evident Scientific, Waltham, MA, USA) equipped with a 20× XLUMPlan FL N objective. Optical stimulation was delivered through the objective using single pulses, pulse trains, or continuous illumination with blue (475 nm) or yellow (590 nm) light, as appropriate for the individual experiments.

For the experiments shown in Figure 2 and Figure 3, recordings were obtained using a loose-apposed, non-sealed pipette configuration. Borosilicate glass pipettes (4–6 MΩ; outer diameter, 1.5 mm; inner diameter, approximately 0.8 mm; King Precision Glass, Claremont, CA, USA) were filled with ACSF and positioned immediately adjacent to the membrane of an identified layer V pyramidal neuron or tdTomato-positive PV-IN. No gigaohm seal was formed, and the pipette contact resistance was maintained below approximately 10 MΩ. Accordingly, the small voltage deflections recorded adjacent to pyramidal neuron somata are interpreted as local extracellular-like, single-cell-associated signals rather than direct measurements of membrane potential. This configuration minimizes intracellular perturbation but does not provide control of membrane potential or intracellular ionic composition. Whole-cell patch-clamp recordings were performed for the experiments shown in Figure 4 and Table 1. For voltage-clamp recordings of inhibitory postsynaptic currents (IPSCs), patch pipettes were filled with an internal solution containing (in mM): 140 Cs-methanesulfonate, 2 MgCl_2_, 10 HEPES, 0.2 EGTA, 2 Na_2_-ATP, and 0.2 Na_2_-GTP. Pyramidal neurons were voltage-clamped at 0 mV for IPSC recordings. For measurements of intrinsic membrane properties, patch pipettes were filled with a potassium-based internal solution containing (in mM): 112.5 K-gluconate, 4 NaCl, 17.5 KCl, 0.5 CaCl_2_, 1 MgCl_2_, 5 ATP (dipotassium salt), 1 NaGTP, 5 EGTA, and 10 HEPES. Cells were initially voltage-clamped at −70 mV to determine passive membrane properties and were subsequently switched to current-clamp mode to measure resting membrane potential (RMP), rheobase, and intrinsic excitability. Rheobase was determined by applying depolarizing current pulses of increasing amplitude from the resting membrane potential until an action potential was elicited. Whole-cell internal solutions were adjusted to pH 7.2 and an osmolarity of 285–290 mOsm. For cell-attached recordings used to monitor PV-IN firing, pipettes contained (in mM): 120 KCl, 11 EGTA, 1 CaCl_2_, 2 MgCl_2_, and 10 HEPES. The solution was adjusted to pH 7.25 with 35 mM KOH (final K+ concentration, 155 mM) and to approximately 310 mOsm with 20 mM glucose. Recordings were performed in I = 0 mode. Electrophysiological signals were recorded using an Axopatch 200B amplifier (Molecular Devices, San Jose, CA, USA), low-pass-filtered at 5 kHz using an 8-pole Bessel filter, digitized at 10 kHz, and acquired and analyzed using EVAN 1.2, a custom LabVIEW-based software package. Importantly, the whole-cell voltage-clamp recordings used to characterize inhibitory currents in Section 3.3 were performed in separate experiments from the loose-apposed recordings presented in Figure 2 and Figure 3.

### 2.6. Statistical Analyses

Animals were randomly assigned to experimental procedures where applicable. Sample sizes were not predetermined by a formal a priori power analysis; animal use was minimized while maintaining sufficient samples for the planned comparisons. The individual animal was the biological experimental unit for behavioral experiments. Across the study, behavioral experiments included 30 mice (18 aged and 12 young), ex vivo electrophysiological experiments included 21 mice (15 aged and 6 young), and in vivo EMG recordings included 12 mice (6 aged and 6 young). Both sexes were included and distributed as evenly as practicable across age groups and experimental cohorts. The overall male-to-female ratio was approximately 2:1. Because several experimental cohorts were small, the study was not powered to evaluate sex-dependent effects. For ex vivo electrophysiology, both the number of animals (N) and the number of recorded cells or slices (n) are reported where applicable. For in vivo EMG recordings, multiple detected EMG events were obtained from individual animals and are considered repeated observations within an animal rather than independent biological replicates. Group/condition information was blinded during data analysis where feasible; however, complete blinding during experimental procedures could not always be maintained because age-related physical differences were apparent in some animals. No animals or experimental units were excluded from the reported analyses unless otherwise stated.

Data are presented as means ± SEM unless otherwise indicated. Comparisons between two independent groups were performed using two-tailed Mann–Whitney tests when assumptions for parametric testing were not met. For analyses containing repeated observations from the same animal, animal identity was treated as the biological experimental unit. For the in vivo EMG analysis, detected events were first averaged within each animal under control and PV-IN photostimulation conditions, so that each mouse contributed one paired value per condition. Animal-level EMG means were then analyzed using a two-way mixed-design repeated-measures ANOVA, with age (young vs. aged) as the between-subject factor and PV-IN photostimulation (control vs. +PVIN stimulation) as the within-subject factor. Main effects of age and photostimulation and the age × photostimulation interaction were reported. Where appropriate, Šídák’s multiple-comparisons test was used for within-age comparisons between control and photostimulation conditions. For EMG experiments, N = 6 animals per age group; the underlying event-level measurements were treated as repeated observations and were not considered independent biological replicates. For the analysis of phasic and tonic IPSCs before and after photostimulation or photoinhibition in whole-cell patch-clamp recordings (Figure 4C,D), the relationship between phasic and tonic currents was evaluated separately before and after light manipulation using linear regression, with the intercept constrained to zero. Each data point represented one pyramidal neuron recorded from a separate brain slice. Statistical significance was defined as *p* < 0.05. The fitted regression slopes were compared using a custom Igor Pro procedure in which the t statistic was calculated from the difference between slopes divided by the standard error of that difference, estimated from the pooled residual variance of the two regressions. Two-sided *p*-values were calculated from Student’s t distribution. The same procedure generated a Hedges’ g-type standardized effect-size estimate for the difference between the fitted slopes, including a small-sample correction; because this implementation differs from the conventional group-mean Hedges’ g calculation, it is reported as a descriptive g-type effect-size estimate. The procedure did not calculate confidence intervals or perform formal regression-assumption diagnostics. Exact *p*-values and F statistics with degrees of freedom are reported where applicable. Analyses were performed using GraphPad Prism 10.4 (GraphPad Software, San Diego, CA, USA) and Igor Pro 8 (Wavemetrics, Lake Oswego, OR, USA), as specified above.

## 3. Results

### 3.1. Aged Mice Exhibit Impaired Motor Coordination but Preserved General Locomotion

In the grid-walking test, we used the three-dimensional detection and analysis method described above. Limb fault number, total travel distance, average speed, and center-to-periphery preference were quantified (Figure 1A–E). No statistically significant differences were detected between aged (N = 18) and young (N = 12) mice for these measures, indicating that general locomotor activity was largely preserved with aging.

In contrast, the rope-pulling test revealed deficits in hindlimb-supported performance in aged mice (Figure 1F–H). Although total rope-pulling distance was lower in aged mice (35.00 ± 2.89 cm) than in young mice (88.00 ± 32.62 cm), the difference did not reach statistical significance. Hindlimb-supported standing time was significantly reduced in aged mice (3.75 ± 0.25 s, N = 4) compared with young mice (10.8 ± 2.35 s, N = 5; *p* = 0.0159, Mann–Whitney test). Given the small cohort, this result is interpreted as evidence of reduced hindlimb-supported performance rather than a direct measure of muscle strength.

The balance beam test further demonstrated age-related deficits in coordination (Figure 1I–K). On the rough-surfaced beam, both age groups were able to traverse the beam, and no significant differences were detected in travel distance or speed. However, on the smooth beam, all aged mice fell within the first minute and were unable to complete the following 2 min testing period, whereas young mice successfully traversed the beam, covering 4539.23 ± 1219.79 mm at an average speed of 81.37 ± 20.45 mm/s. Travel distance and speed were significantly reduced in aged mice (*p* = 0.0023 and *p* = 0.001, respectively, Mann–Whitney test).

Together, these behavioral results indicate that aging produces marked impairments in motor coordination and balance while leaving general locomotor activity relatively intact.

### 3.2. Light-Evoked Extracellular-like Voltage Responses Are Detected at Pyramidal Neuron Somata in Aged M1 Cortex

To examine age-related differences in network responses associated with PV-IN activation, we performed loose-apposed, non-sealed recordings in acute M1 slices from young and aged PV-Cre/Ai27 mice. The recording pipette was positioned immediately adjacent to the somatic membrane without forming a gigaohm seal, and the contact resistance was maintained below approximately 10 MΩ. Accordingly, these recordings are best considered local extracellular-like, single-cell-associated voltage recordings rather than conventional whole-cell or sealed cell-attached measurements of membrane potential. PV-INs were identified by tdTomato fluorescence and robust light-evoked firing. Putative pyramidal neurons were selected based on their layer V location and characteristic pyramidal morphology. Limitations regarding cell identification and reporter specificity are addressed in the Section 4.

In slices from aged mice, a single 5 ms blue-light pulse consistently produced a reproducible negative-going voltage deflection recorded adjacent to the soma of layer V pyramidal neurons (Figure 2A). In contrast, no light-evoked response exceeding the predefined detection threshold was detected under the same recording configuration in the young cohort. To maintain consistency across recordings, the pipette was positioned adjacent to the somatic membrane for all cells. Responses from proximal-axonal or -dendritic compartments were not examined in the present study and may exhibit different signal characteristics.

Light-evoked responses were detected and analyzed using EVAN software. A response was considered detectable when its amplitude exceeded approximately two times the standard deviation (SD) of the pre-stimulus baseline. Additional detection parameters included a baseline window of 100 data points, a detection length of 0.1 ms, and a dead time of 2 ms. Because membrane potential and intracellular ionic composition were not controlled in this loose-apposed configuration, the recorded voltage deflections are not interpreted as direct measurements of postsynaptic membrane potential.

In aged slices, detectable responses had an average extracellular-like amplitude of 0.32 ± 0.01 mV, a 10–90% rise time of 1.20 ± 0.08 ms, a rise rate of 0.23 ± 0.02 V/s, and a decay time constant of 2.45 ± 0.25 ms (n = 24 neurons from 7 aged mice; Figure 2C–F). In comparison, recordings from eight neurons obtained from four young mice showed no light-evoked responses exceeding the predefined detection threshold. Baseline standard deviation (SD) was used to quantify the recording noise level and did not differ significantly between young- and aged-slice recordings (Figure 2G), indicating that the absence of detectable light-evoked responses in young slices was not attributable to a higher baseline noise level.

To determine the transmitter dependence of the light-evoked response observed in aged M1, we first applied the GABA_A_ receptor antagonist gabazine (GBZ; 40 µM). The response persisted during GABA_A_ receptor blockade and increased in magnitude and duration. Subsequent addition of the ionotropic glutamate receptor antagonists DNQX (10 µM) and APV (50 µM) abolished the light-evoked response (Figure 3A). These pharmacological findings demonstrate that the response engaged during PV-IN-targeted photostimulation requires ionotropic glutamatergic transmission. However, these experiments do not establish the underlying synaptic topology and cannot distinguish among recurrent excitation, disinhibition, or other polysynaptic network mechanisms.

Recordings from tdTomato-labeled PV-INs confirmed that the same photostimulation reliably triggered action-potential firing under the tested pharmacological conditions (Figure 3B,C). Together, these observations establish a temporal association between PV-IN photostimulation and the glutamate-dependent extracellular-like network response detected near layer V pyramidal neuron somata in aged M1. Importantly, these findings are interpreted as evidence for recruitment of a glutamatergic network mechanism, rather than evidence for a monosynaptic excitatory connection from PV-INs to pyramidal neurons.

### 3.3. PV-INs Maintain Inhibitory Coupling with Pyramidal Neurons During Prolonged Stimulation in the Aged Motor Cortex

To investigate inhibitory connectivity between PV-INs and pyramidal neurons in aged M1, we examined inhibitory synaptic transmission during repetitive optogenetic manipulation of PV-IN activity. The loose-apposed experiments described above identified a glutamate-dependent network response during PV-IN-targeted photostimulation but did not establish a direct excitatory synapse. The experiments in this section therefore addressed a separate question: whether conventional inhibitory PV-IN-to-pyramidal transmission remained detectable in aged M1.

To further characterize this interaction, whole-cell patch-clamp recordings were obtained from pyramidal neurons to monitor postsynaptic currents, while cell-attached recordings were performed from PV-INs to verify their responsiveness to sustained photostimulation. Brain slices were stimulated with either single-pulse or paired-pulse blue-light trains (2 ms inter-pulse interval) delivered at 20 Hz.

Simultaneous recordings revealed a strong temporal coupling between PV-IN firing and inhibitory synaptic responses in pyramidal neurons. Single-pulse photostimulation reliably evoked action potentials in PV-INs and generated corresponding IPSCs in pyramidal neurons, demonstrating robust inhibitory transmission from PV-INs to pyramidal cells (Figure 4A). During paired-pulse stimulation, PV-INs faithfully followed both light pulses throughout the stimulation train, and pyramidal neurons exhibited IPSCs containing distinct primary and secondary peaks that mirrored the paired-pulse activation pattern (Figure 4B). These findings indicate that PV-INs retain the capacity to reliably drive inhibitory synaptic transmission during prolonged repetitive activation in the aged motor cortex.

We next examined whether sustained manipulation of PV-IN activity altered the relationship between phasic and tonic inhibitory currents. Because eNpHR3.0 is a chloride pump, sustained illumination can alter intracellular chloride and thereby influence GABA_A_ receptor-mediated currents after light offset. Following 10 s of 20 Hz PV-IN photostimulation with ChR2, the fitted relationship between phasic and tonic currents remained similar before and after stimulation (zero-intercept slope: 0.0361 ± 0.0267 before vs. 0.0391 ± 0.0218 after photostimulation; slope comparison, t(10) = 0.080, two-sided *p* = 0.938; Hedges’ g-type effect-size estimate = 0.069; Figure 4C). Following 10 s of PV-IN photoinhibition with eNpHR3.0, the fitted slope decreased from 0.1734 ± 0.0876 before photoinhibition to 0.1031 ± 0.0938 after it; however, the difference was not statistically significant (t(8) = 0.490, two-sided *p* = 0.637; Hedges’ g-type effect-size estimate = 0.416; Figure 4D). We therefore interpret the post-photoinhibition change descriptively and do not consider it definitive evidence of a compensatory network mechanism.

### 3.4. Activation of M1 PV-INs Enhances CST-Mediated Motor Output in Aged Mice

Layer V pyramidal neurons in M1 provide the major cortical origin of the CST. Their axons descend to the spinal cord and terminate within spinal gray matter, where they influence hindlimb motor output through spinal interneuron, premotor, and motoneuron-related circuits. Because aging was associated with selective deficits in balance and hindlimb-supported performance, we next investigated whether local PV-IN activity alters M1-evoked corticospinal motor output in aged mice.

Whole-cell recordings were obtained from retrogradely labeled CST-projecting neurons and neighboring unlabeled layer V pyramidal neurons in aged mice. A representative image of a recorded retrogradely labeled neuron and a neighboring unlabeled pyramidal neuron is shown in Figure 5D. Within this limited sample (n = 5 cells per group from N = 5 mice), no statistically detectable differences were identified in resting membrane potential, input resistance, membrane capacitance, membrane time constant, or rheobase (Table 1). Because of the small sample size, these findings should not be interpreted as evidence of equivalence, and moderate group differences cannot be excluded.

We next examined the functional consequences of PV-IN activation on M1-evoked hindlimb motor output in vivo. Electrical stimulation was delivered to the hindlimb representation area of M1 while EMG responses were recorded from the contralateral tibialis anterior (TA) muscle. The TA is innervated by peripheral motoneurons and is strongly recruited by descending motor pathways; thus, it provides a readout of hindlimb motor output influenced by corticospinal signaling rather than a muscle directly innervated by CST axons. Responses were compared under baseline conditions and during optogenetic activation of local PV-INs.

Representative recordings are shown in Figure 6A,B. Animal identity was treated as the biological experimental unit, and repeated EMG events from the same animal were not considered independent biological replicates. Event measurements were averaged within each mouse for the control and PV-IN stimulation (+PV) conditions before statistical analysis (N = 6 young and N = 6 aged mice). PV-IN photostimulation produced an age-dependent effect on M1-evoked EMG amplitude, demonstrated by a significant age × photostimulation interaction (F(1,10) = 8.09, *p* = 0.0174). The main effect of photostimulation was also significant (F(1,10) = 8.27, *p* = 0.0165), whereas the main effect of age was not (F(1,10) = 2.22, *p* = 0.1675). Mean EMG amplitude was essentially unchanged by PV-IN photostimulation in young mice (control, 0.0775 ± 0.0137 mV; +PV, 0.0778 ± 0.0124 mV), whereas an increase was observed in aged mice (control, 0.0842 ± 0.0136 mV; +PV, 0.1366 ± 0.0246 mV). Šídák-adjusted post hoc comparisons detected no differences in young mice (*p* = 0.9977); the within-aged comparison showed a trend toward increased amplitude but did not reach the adjusted significance threshold (*p* = 0.0621) (Figure 6C). Thus, the significant interaction, rather than the within-group post hoc comparison, supports an age-dependent influence of PV-IN activation on EMG amplitude.

Animal-level analysis of the secondary EMG parameters revealed no significant age × photostimulation interaction for event duration (F(1,10) = 2.17, *p* = 0.1712); neither the main effect of age (F(1,10) = 1.96, *p* = 0.1916) nor photostimulation (F(1,10) = 2.80, *p* = 0.1251) reached significance (Figure 6D). For IEI, there was a significant main effect of age (F(1,10) = 8.22, *p* = 0.0167) but no main effect of photostimulation (F(1,10) = 0.01, *p* = 0.9137) and no age × photostimulation interaction (F(1,10) = 2.04, *p* = 0.1836) (Figure 6E). The event frequency parameter showed a significant main effect of age (F(1,10) = 15.43, *p* = 0.0028) and a significant age × photostimulation interaction (F(1,10) = 6.08, *p* = 0.0334), whereas the main effect of photostimulation was not significant (F(1,10) = 0.02, *p* = 0.9030) (Figure 6F). Šídák-adjusted within-age comparisons for frequency were not significant in either young (*p* = 0.1081) or aged mice (*p* = 0.3632). Accordingly, these secondary measures are interpreted primarily in terms of the factorial effects rather than isolated within-group comparisons (Table 2).

To further evaluate the temporal organization of motor output, we quantified burstiness and memory of the detected EMG events. Burstiness measures the tendency of events to cluster in time, whereas memory reflects correlations between consecutive inter-event intervals. Although the distribution of burstiness values in aged mice appeared shifted toward lower values compared with young animals, neither burstiness nor memory differed significantly between age groups or between control and PV-IN activation conditions (Figure 6G,H). Thus, the detected temporal organization of EMG activity was not significantly altered by PV-IN photostimulation under the conditions tested.

Together, these findings indicate that PV-IN photostimulation has an age-dependent influence on M1-evoked hindlimb motor output. Although the increased response in aged animals is compatible with altered recruitment of corticospinal-related motor circuits, the present experiments do not establish that this change is compensatory or beneficial; increased cortical output could also reflect dysregulated excitatory–inhibitory network function.

## 4. Discussion

The principal finding of this study is that aging alters the functional influence of PV-IN-associated networks on layer V pyramidal neurons and motor-cortical output. In aged but not young M1 slices, photostimulation of the PV-IN-targeted circuit elicited reproducible extracellular-like local voltage responses near layer V pyramidal neuron somata. These responses persisted and increased during GABA_A_ receptor blockade but were abolished by DNQX and APV, indicating a requirement for ionotropic glutamatergic transmission. In separate whole-cell experiments, conventional inhibitory PV-IN-to-pyramidal transmission remained readily detectable during repetitive PV-IN activation. Together, these observations argue against a simple conversion of PV-IN signaling from inhibitory to excitatory and instead support an age-dependent change in the broader cortical network recruited by PV-IN activity.

Several circuit mechanisms could contribute to this age-dependent response. PV-IN activation may indirectly recruit recurrent excitation, alter interactions among inhibitory interneuron populations to produce disinhibition, or engage excitatory circuits whose recruitment threshold changes with aging. Aging is associated with alterations in GABAergic synaptic function, interneuron properties, cortical microcircuit organization, and PV-IN/perineuronal-net integrity [20,21,22]. More generally, PV-INs are key regulators of cortical timing, synchrony, and excitation/inhibition balance [23,24,25], so changes in their network interactions could substantially alter how activity propagates through M1. The enhancement of the extracellular-like response during GABA_A_ receptor blockade further supports an interaction between inhibitory and excitatory network components rather than a change in PV-IN transmitter phenotype. Thus, our findings are most consistent with age-dependent reorganization of PV-IN-associated network interactions, although the precise synaptic pathway remains to be determined.

Importantly, age-related changes in PV-IN function do not necessarily reflect a generalized loss of PV interneurons. Across multiple mouse cortical regions, PV-neuron density can remain stable with aging despite reductions in PV expression and changes in perineuronal-net organization [22]. Similarly, analysis of mouse visual cortex from 3 to 24 months found no substantial loss of inhibitory neurons and largely preserved representation of PV and other major interneuron subtypes, despite pronounced age-related simplification and reduced structural plasticity of interneuron arbors [26]. These observations support the possibility that the age-dependent effects observed here arise from the functional and circuit-level remodeling of surviving PV-IN networks rather than simply from loss of PV-INs.

Changes in chloride homeostasis could further influence inhibitory network function during aging. GABAergic efficacy depends strongly on intracellular chloride regulation by cation-chloride cotransporters, particularly KCC2 and NKCC1 [27,28]. Disruption of chloride extrusion can weaken hyperpolarizing inhibition and alter neuronal excitability. Although the glutamate-antagonist experiments demonstrate that ionotropic glutamatergic transmission is required for the extracellular-like response observed in aged M1, altered chloride regulation could still modify the excitability of the surrounding network. This issue is particularly relevant to the eNpHR3.0 experiments because prolonged halorhodopsin activation can increase intracellular chloride and produce post-illumination changes in excitability [29]. Accordingly, the rebound increase in phasic IPSC activity following prolonged PV-IN photoinhibition is reported descriptively and should not be interpreted as direct evidence of a compensatory network mechanism.

The age-dependent network change observed ex vivo was accompanied by an altered effect of PV-IN activation on descending motor output in vivo. Layer V M1 pyramidal neurons provide a major cortical origin of the CST, which influences limb movement largely through spinal interneuronal and premotor circuits [7,8]. Consistent with this organization, retrograde labeling from lumbar spinal segments L2-L4 identified CST-projecting layer V pyramidal neurons in M1. In aged mice, activation of M1 PV-INs increased M1-evoked EMG responses recorded from the contralateral tibialis anterior, whereas little change was observed in young mice. These findings extend the slice observations by showing that the age-dependent alteration in PV-IN-associated circuitry is also evident at the level of descending motor output.

The behavioral phenotype provides additional functional context for these physiological findings. Aged mice showed relatively preserved general locomotor performance in the grid-walking assay but greater impairment in tasks placing higher demands on balance, postural stability, and hindlimb-supported performance, consistent with the selective deterioration of motor control reported with aging [14]. This pattern suggests that aging did not uniformly suppress motor activity but preferentially affected more demanding aspects of motor function. Nevertheless, the present experiments do not establish a direct causal link between the identified cortical network response and the behavioral deficits.

The functional significance of the observed reorganization remains open to more than one interpretation. Increased recruitment of cortical and descending motor output could help maintain motor commands as other components of the aging motor system become less efficient. Alternatively, enhanced output could reflect reduced inhibitory precision or dysregulated excitation/inhibition balance and thereby contribute to impaired motor coordination. Our findings do not distinguish between these possibilities. Rather than defining the observed remodeling as inherently adaptive or maladaptive, the present results indicate that the relationship among PV-IN activity, local excitatory networks, and corticospinal output is substantially altered with aging.

Taken together, our findings identify age-dependent reorganization of PV-IN-associated motor-cortical circuitry across multiple functional levels. Conventional inhibitory PV-IN-to-pyramidal transmission remains detectable, while PV-IN-targeted stimulation in aged M1 additionally recruits an ionotropic glutamate receptor-dependent network response and exerts a greater facilitatory influence on M1-evoked hindlimb motor output. These results support the view that aging changes how inhibitory interneuron activity is integrated within local excitatory networks and translated into descending motor output.

## 5. Limitations

Several limitations should be considered when interpreting these findings. First, the responses in Figure 2 and Figure 3 were recorded using a loose-apposed, non-sealed configuration and therefore represent local extracellular-like voltage signals rather than direct measurements of postsynaptic membrane potential. Although baseline noise was comparable between age groups and responses were defined using a predefined detection threshold, matched whole-cell recordings would provide more direct characterization of the underlying membrane response. Second, quantitative histological validation of PV/ChR2 colocalization and potential ectopic reporter expression was not available in the present study. Because quantitative histological validation of reporter specificity and PV-IN density was not performed in the present cohorts, low-level ectopic reporter expression or age-related differences in PV-IN density cannot be fully excluded. Accordingly, the present findings are interpreted as reflecting a PV-IN-targeted network response rather than a definitive direct PV-IN synaptic mechanism. Future studies incorporating quantitative PV/tdTomato colocalization, excitatory-marker exclusion, and PV-IN density measurements in young and aged M1 will be important to further establish cellular specificity. Third, several experimental cohorts were relatively small, and the 14–24-month aged group encompasses a physiologically heterogeneous period; consequently, the study was not sufficiently powered to resolve sex- or precise-age-dependent effects. Fourth, the in vivo experiments were performed under anesthesia, and TA EMG represents a downstream measure of descending motor output rather than direct CST activity. Finally, the pharmacological experiments establish the involvement of ionotropic glutamatergic transmission but do not resolve the underlying synaptic topology, and the present study does not establish a causal relationship between the physiological network changes and age-related behavioral deficits. These questions will require pathway-specific and behaviorally linked experiments in future studies.

## 6. Conclusions

Aging is associated with reorganization of PV-IN-associated network function in M1. Conventional inhibitory PV-IN-to-pyramidal transmission remains detectable, whereas PV-IN-targeted photostimulation in aged cortex additionally engages an ionotropic glutamate receptor-dependent network response and increases M1-evoked hindlimb motor output. These findings indicate that aging alters the integration of inhibitory and excitatory network activity within motor cortex and its influence on descending motor output. Whether this reorganization contributes to compensation, circuit dysfunction, or both under different behavioral conditions remains to be determined.

## Figures and Tables

**Figure 1:**
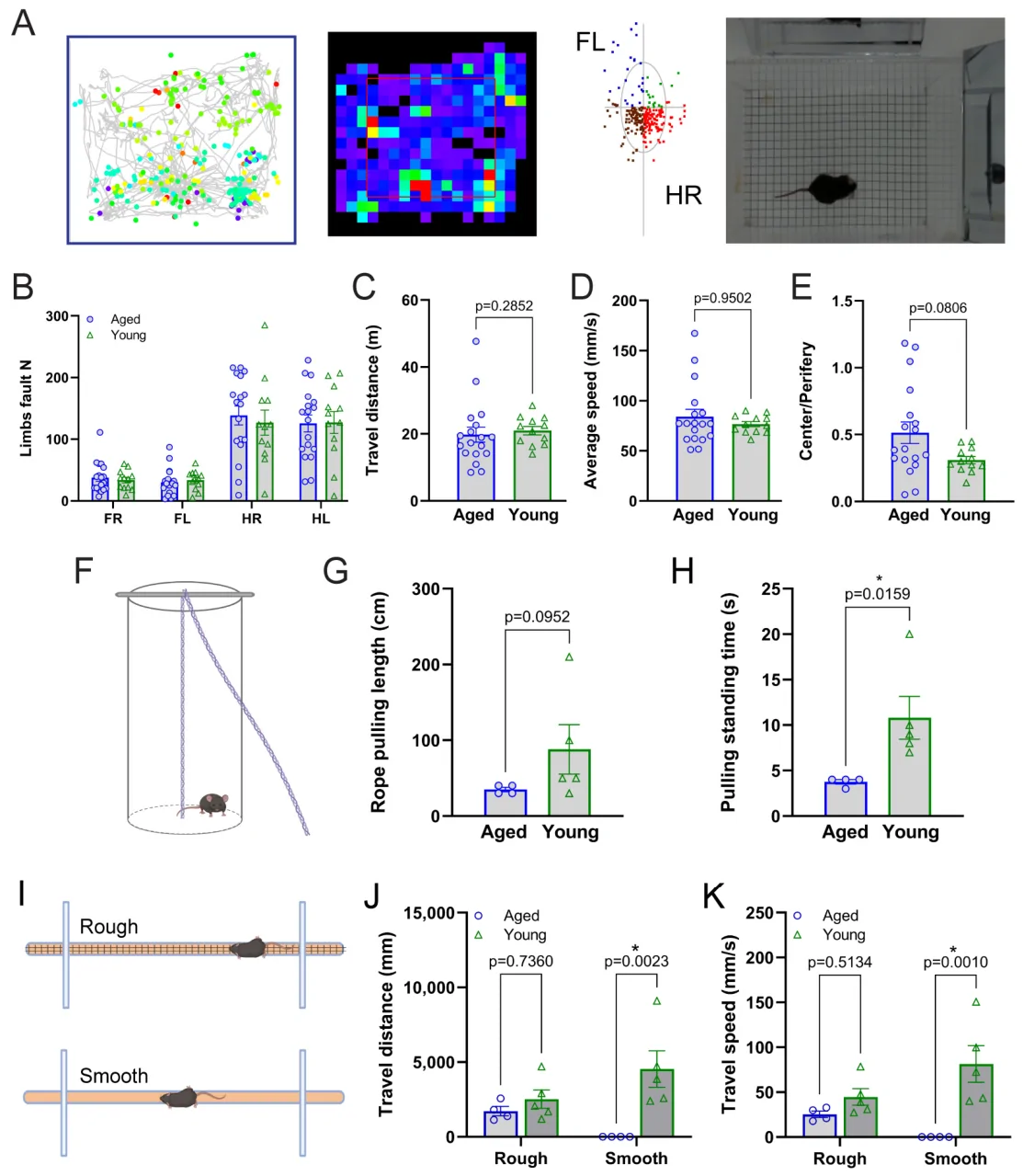
Behavioral assessment of locomotor and motor coordination in young and aged mice. (**A**–**E**) Grid-walking test. (**A**) Representative overview of the grid-walking assay and analysis. From left to right: representative locomotor trajectory (gray) recorded during the 10 min test; color-coded markers indicating limb faults and nose-reaching events beyond the grid; heat map showing the preferred occupancy of the arena, with the center region outlined in red and the surrounding area defined as the periphery; and schematic illustrations of limb fault scoring from head-up and rear-view perspectives. A top-view image of the grid-walking apparatus is also shown, consisting of a mesh-bottom walking box (20 cm × 30 cm base and 20 cm heigh transparent acrylic walls 5 mm in thickness) with two mirrors positioned at approximately 45° beneath the apparatus to facilitate the visualization and scoring of limb placement errors. (**B**–**E**) Quantification of grid-walking performance in young (in green) and aged (in blue) mice, including (**B**) total limb fault number, (**C**) total travel distance (m), (**D**) average locomotor speed (mm/s), and (**E**) center-to-periphery ratio during the 10 min testing period. No significant differences were observed between young and aged mice in any of these locomotor parameters. (**F**–**H**) Rope-pulling test. (**F**) Representative illustration of the rope-pulling apparatus. Quantification of (**G**) the total rope length pulled during the 2 min testing period (cm) and (**H**) cumulative hindlimb standing time (s) while pulling the rope. Young mice spent significantly more time standing on their hindlimbs during rope-pulling behavior than aged mice. (**I**–**K**) Balance beam test. (**I**) Representative images of the balance beam apparatus consisting of two beams with different surface textures (upper, rough surface; lower, smooth surface). Quantification of (**J**) total travel distance and (**K**) average travel speed (mm/s) on the beam during the 2 min test period. Statistical significance is indicated as labeled in the figures. The young mice group is shown in green, and the aged mice group is shown in blue. *: *p* ≤ 0.05.

**Figure 2:**
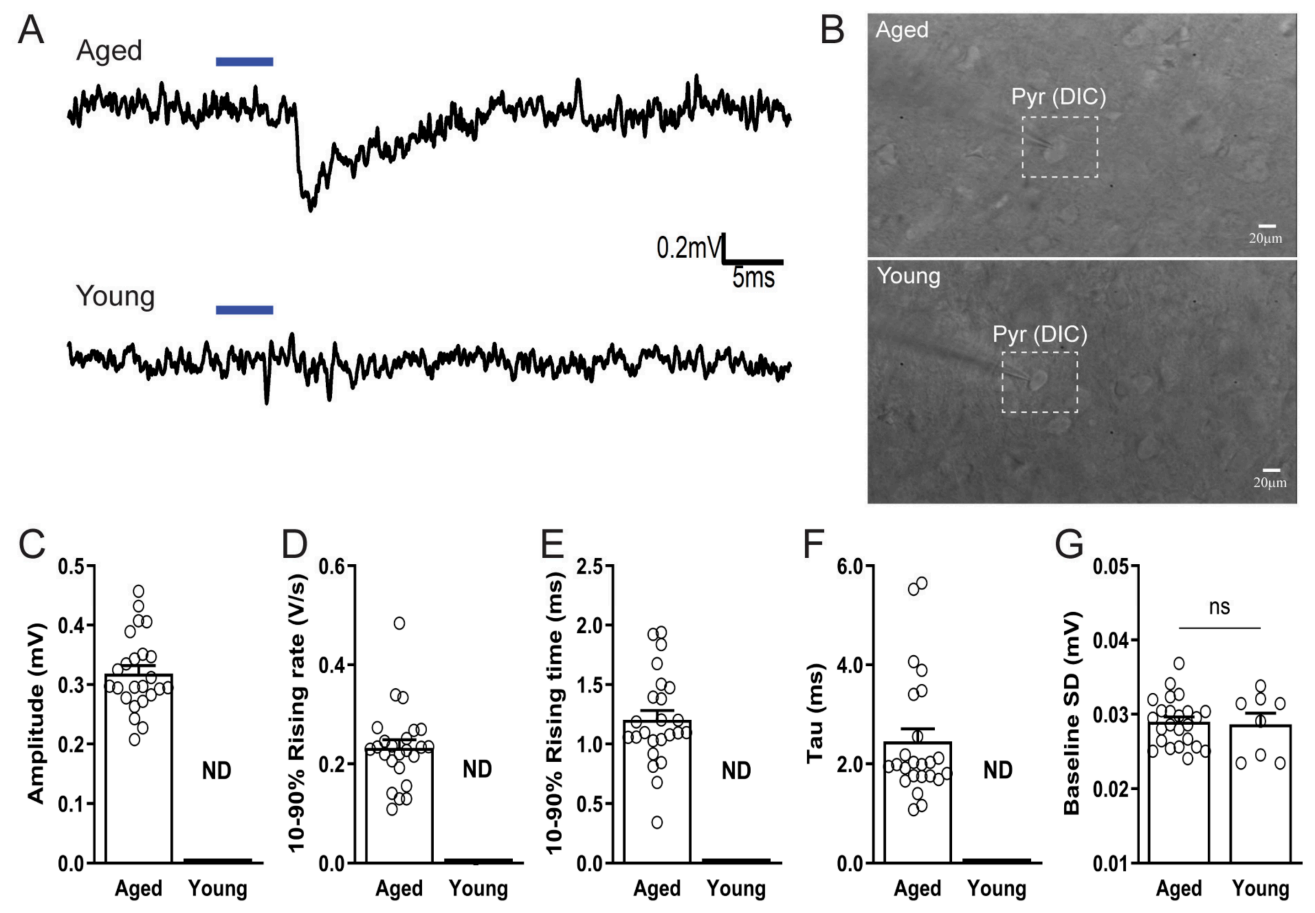
Loose-apposed extracellular-like voltage responses associated with optogenetic stimulation of M1 layer V PV interneurons. (**A**) Representative local voltage traces obtained using a non-sealed recording pipette positioned immediately adjacent to the soma of layer V pyramidal neurons. A reproducible light-evoked voltage deflection was detected in aged slices, whereas no response exceeding the predefined detection threshold was detected under the same recording configuration in young slices. The blue bar indicates the 5 ms blue-light stimulus. Because no gigaohm seal was formed and neither membrane potential nor intracellular ionic composition was controlled, these signals are interpreted as local extracellular-like voltage responses rather than direct measurements of postsynaptic membrane potential. (**B**) Representative IR-DIC images showing the loose-apposed recording configuration at layer V pyramidal neuron somata in aged and young M1 slices. Representative DIC image acquired using a 20× water-immersion objective. Scale bar = 20 µm. (**C**–**F**) Quantitative characteristics of the light-evoked responses detected in aged slices, including (**C**) extracellular-like response amplitude, (**D**) 10–90% rise time, (**E**) 10–90% rise rate, and (**F**) decay time constant. No light-evoked responses exceeding the predefined detection threshold were detected in young slices (ND). (**G**) Baseline standard deviation (SD), used as a measure of recording noise, in recordings from aged and young slices. No significant difference in baseline SD was detected between the two age groups. ND, no response detected above the predefined threshold. ns: not significant, *p* > 0.05.

**Figure 3:**
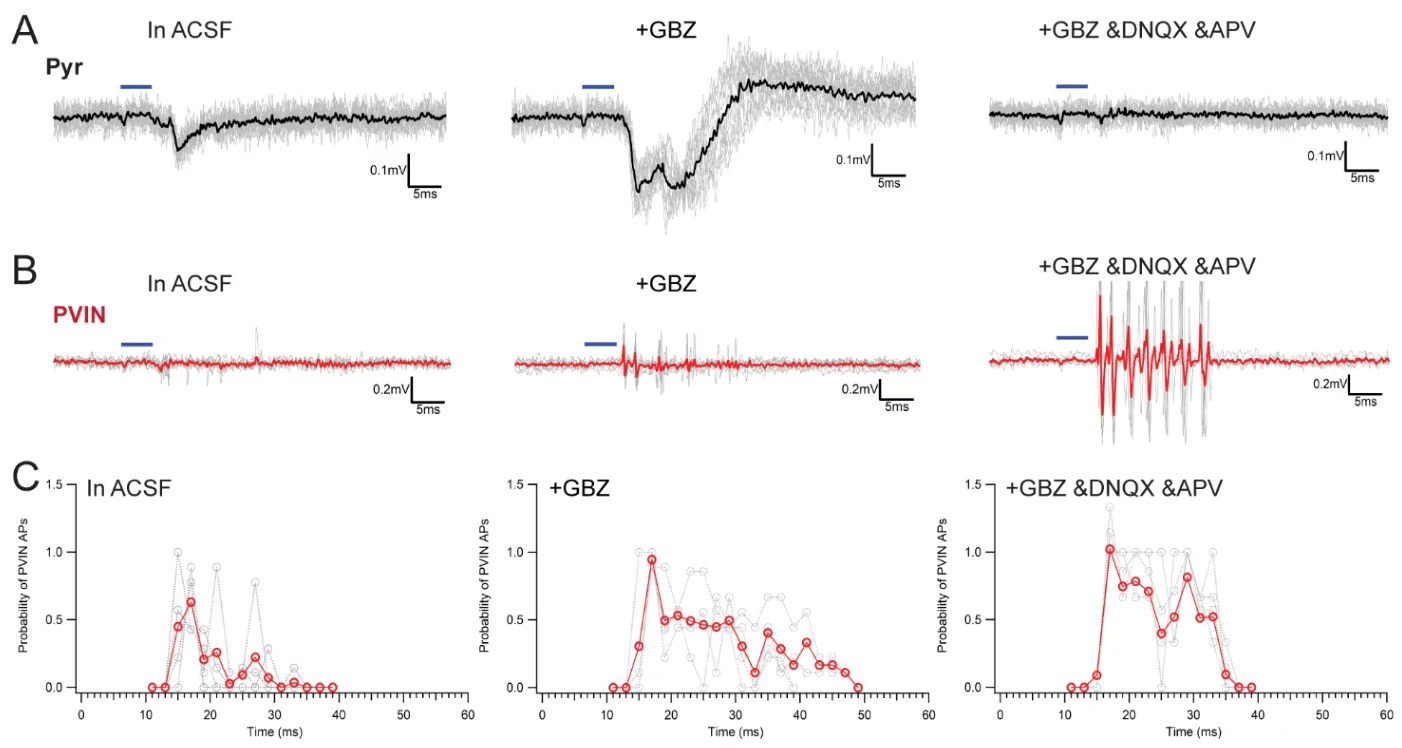
Light-associated extracellular-like responses in aged M1 depend on ionotropic glutamatergic transmission and occur during reliable PV interneuron firing. (**A**) Representative local voltage responses recorded near layer V pyramidal neurons in aged slices under ACSF, ACSF + 40 µM gabazine (GBZ), and ACSF + 40 µM GBZ + 10 µM DNQX + 50 µM APV. The response persisted during GABA_A_ receptor blockade but was abolished after addition of DNQX and APV. (**B**) Representative light-evoked action potential firing from tdTomato-labeled PV interneurons recorded in the loose-apposed configuration under the same pharmacological conditions. The black trace in A and the red trace in B represent the averages of five individual trials (shown in gray) recorded from the same cell. The blue bars in A and B indicate 5 ms light pulse stimulation. (**C**) Action potential probability as a function of time after blue-light stimulation. Spike occurrence was analyzed at approximately 2 ms temporal resolution; n = 4 slices from N = 4 animals. The red trace in C represents the average of all individual recordings from each animal (shown in gray). These data support a PV-IN-engaged glutamatergic network mechanism but do not resolve whether the excitatory recruitment is mono- or polysynaptic.

**Figure 4:**
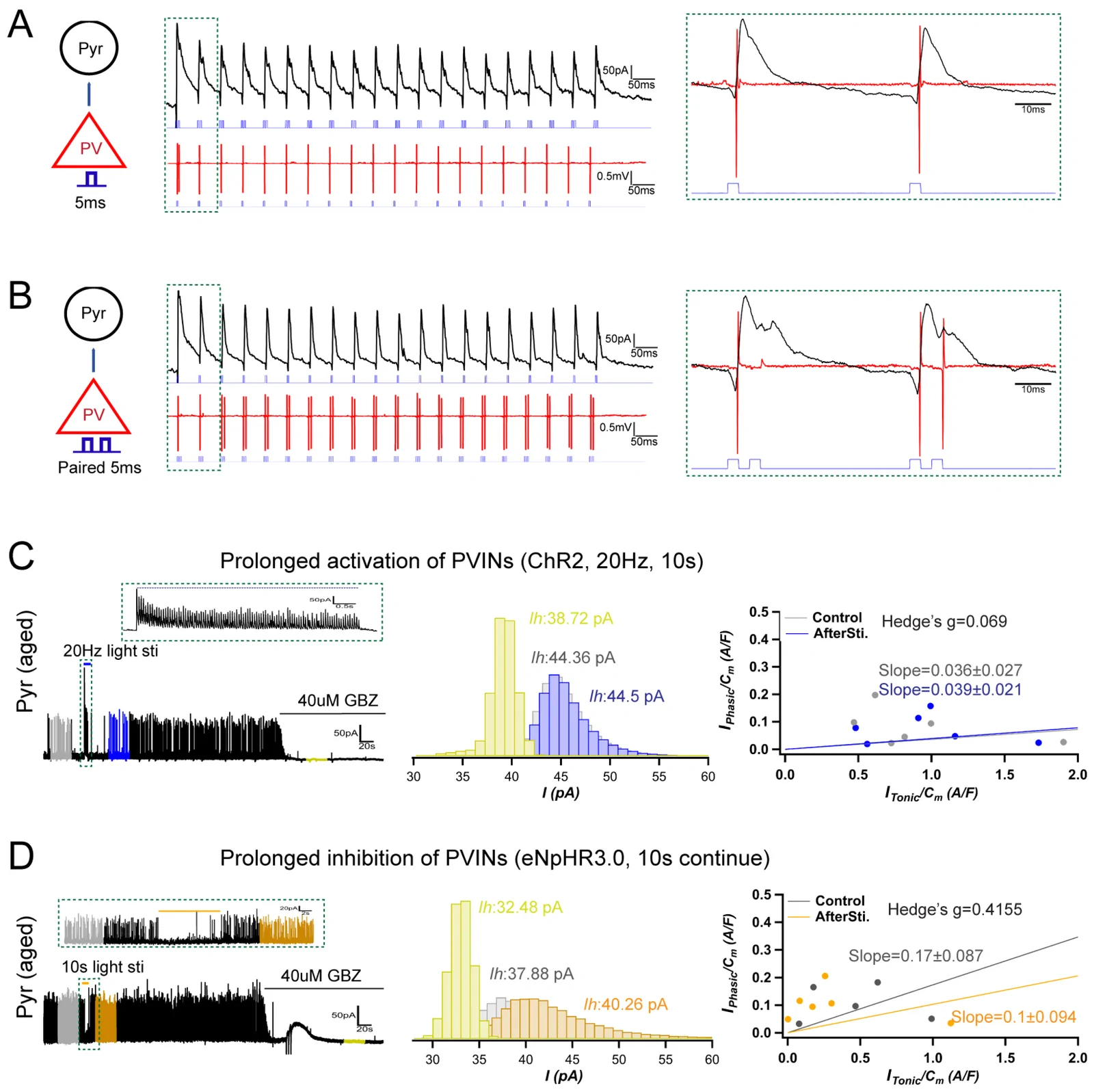
Functional coupling between PV interneurons and pyramidal neurons in the aged motor cortex during prolonged optogenetic stimulation. (**A**) A 20 Hz train of single blue-light pulses (5 ms pulse duration, 1 s) reliably evoked action potentials in PV interneurons (red) and corresponding inhibitory postsynaptic currents (IPSCs) in layer V pyramidal neurons (black), demonstrating effective inhibitory synaptic transmission. The temporal relationship among photostimulation, PV interneuron firing, and IPSCs is summarized on the right. (**B**) A 20 Hz paired-pulse stimulation protocol (5 ms pulse duration, 2 ms inter-pulse interval, 1 s) reliably elicited action potential firing in PV interneurons and generated IPSCs with distinct primary and secondary peaks corresponding to the first and second light pulses, respectively. The timing relationships between photostimulation, PV interneuron firing, and IPSCs are illustrated on the right. (**C**) Effects of prolonged PV-IN activation on the relationship between phasic and tonic inhibitory currents before and after 10 s of continuous photostimulation. (**D**) Effects of prolonged PV-IN photoinhibition on the relationship between phasic and tonic inhibitory currents before and after 10 s of continuous eNpHR3.0 activation. For (**C**,**D**), representative traces are shown on the left, while the middle and right panels summarize the phasic-tonic current zero-intercept regression slopes: 0.1734 ± 0.0876 before and 0.1031 ± 0.0938 after photoinhibition (slope comparison, t(8) = 0.490, two-sided *p* = 0.637; Hedges’ g-type effect-size estimate = 0.416). Zero-intercept linear-regression slopes were 0.0361 ± 0.0267 before and 0.0391 ± 0.0218 after photostimulation (slope comparison, t(10) = 0.080, two-sided *p* = 0.938; Hedges’ g-type effect-size estimate = 0.069). Each data point represents one pyramidal neuron recorded from a separate brain slice. Regression slopes were compared using the custom Igor Pro procedure described in Statistical Analyses; the reported Hedges’ g-type values are descriptive standardized effect-size estimates generated by that procedure rather than conventional group-mean Hedges’ g values.

**Figure 5:**
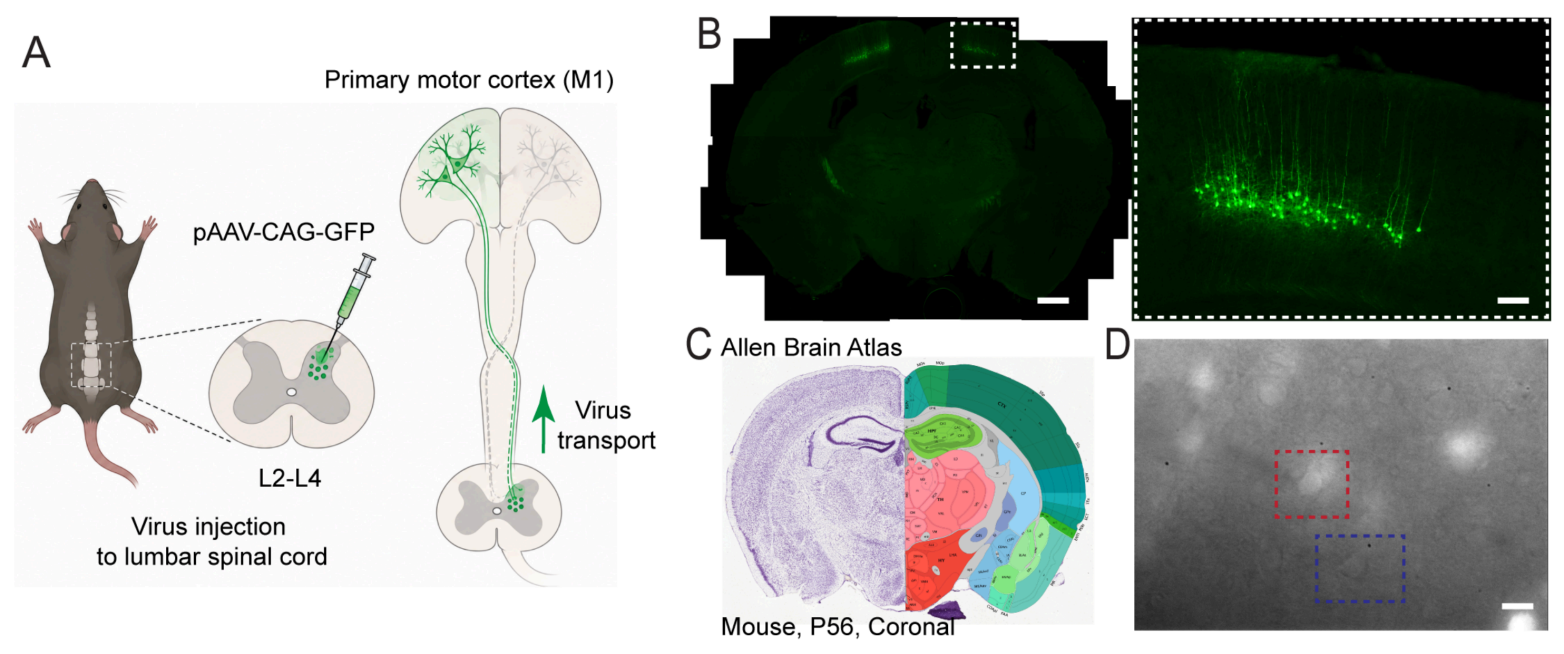
Retrograde labeling and electrophysiological identification of CST-projecting pyramidal neurons. (**A**) Schematic of retrograde viral-tracer injection into the lumbar spinal cord (L2–L4) to label CST-projecting pyramidal neurons in primary motor cortex (M1). (**B**) Representative images showing the distribution of retrogradely labeled cortical neurons at 4× (left; scale bar = 100 µm) and 10× (right; scale bar = 10 µm) magnification. (**C**) Corresponding coronal section from the Allen Mouse Brain Atlas indicating the cortical region containing the retrogradely labeled neurons. Image source: Allen Mouse Brain Atlas (mouse, P56, coronal section). (**D**) Representative image of a patch-clamp recording from a retrogradely labeled CST-projecting pyramidal neuron (red frame; recording electrode visible) and a neighboring unlabeled pyramidal neuron (blue frame). Scale bar = 20 µm.

**Figure 6:**
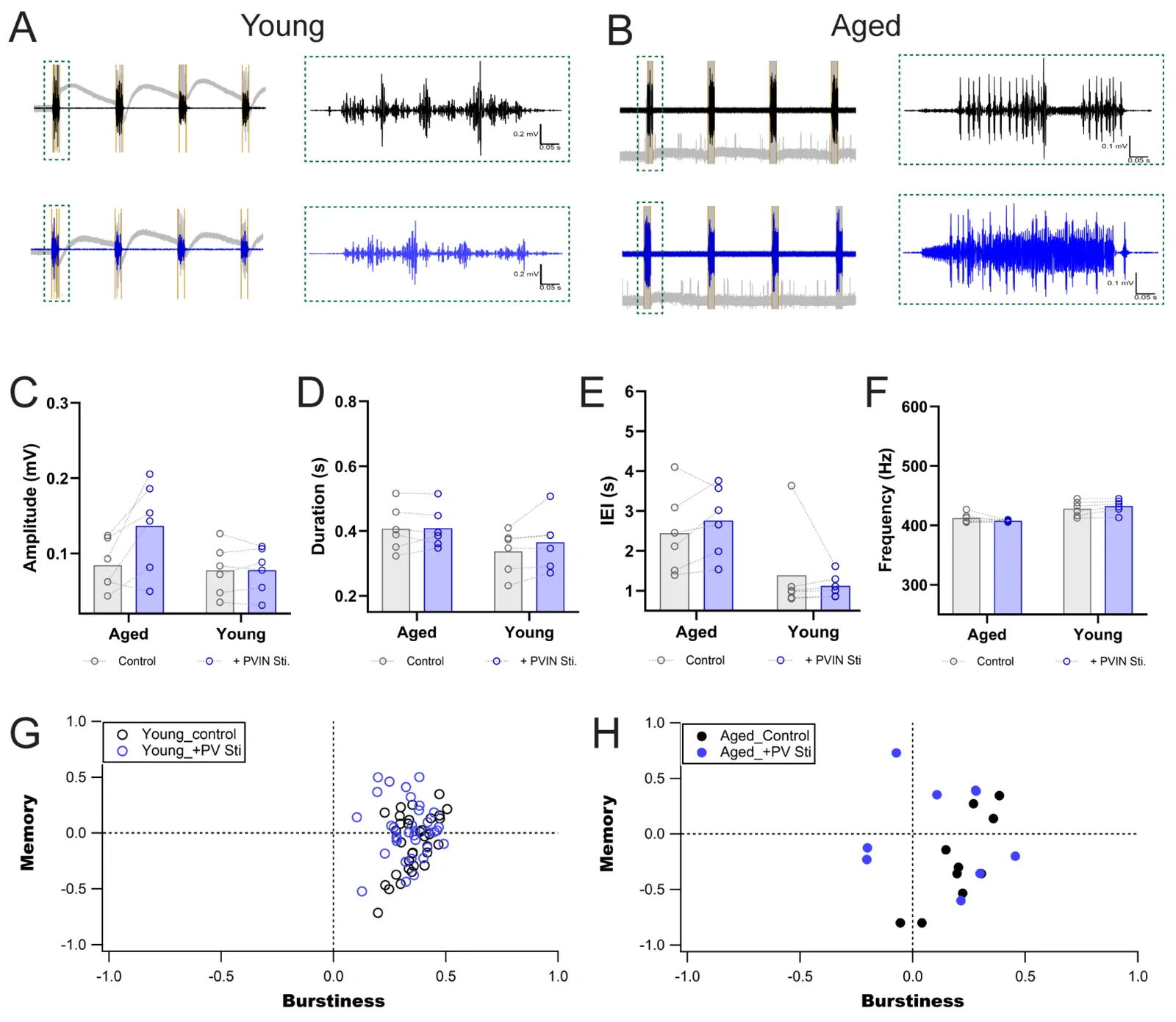
Age-dependent effects of M1 PV interneuron activation on M1-evoked hindlimb EMG activity. (**A**,**B**) Representative raw and filtered electromyography (EMG) recordings. Gray traces show the raw EMG signals, whereas black (no light stimulation) and blue (light stimulation) traces represent EMG signals band-pass-filtered between 200 and 2000 Hz. Enlarged views of representative filtered EMG event clusters are shown on the right. The upper panels illustrate recordings obtained without optogenetic stimulation of M1 PV interneurons, whereas the lower panels show recordings during blue-light activation of PV interneurons. Yellow binary traces indicate event-detection windows identified by the automated detection algorithm. (**C**–**F**) Animal-level analysis of EMG parameters under control conditions and during optogenetic activation of M1 PV interneurons, including (**C**) average event amplitude, (**D**) event duration, (**E**) inter-event interval (IEI), and (**F**) event frequency parameter. Multiple detected events were averaged within each mouse for each condition before statistical analysis; N = 6 young and N = 6 aged mice. Statistics were performed using a two-way mixed-design repeated-measures ANOVA with age as the between-subject factor and photostimulation as the within-subject factor, followed by Šídák’s multiple-comparisons test where appropriate. (**G**,**H**) Burstiness–memory analysis of EMG activity. Scatter plots illustrate the distributions of burstiness and memory under control conditions and during optogenetic activation of M1 PV interneurons. Values are presented as means ± SEM unless otherwise indicated.

**Table 1:** Intrinsic membrane properties of CST-projecting and unlabeled pyramidal neurons in the aged M1 cortex.

	n	Cm (pF)	Rm (MΩ)	Tau (ms)	RMP (mV)	Rheobase (pA)
**PYR (unlabeled)**	5	106.9 ± 23.1	255.6 ± 33.4	2.18 ± 0.5	−71.64 ± 0.8	326 ± 42.6
**PYR (retro)**	5	159.8 ± 16.2	195.6 ± 25.2	3.44 ± 0.4	−68.12 ± 1.9	327 ± 55.9
**Mann–Whitney test (*p*)**		0.1508	0.2222	0.0794	0.1349	0.6905

Values are presented as means ± SEM. Exact *p*-values are shown in the bottom row. No statistically detectable differences were identified in this limited sample; however, the sample size does not permit exclusion of moderate effects. n = 5 cells per group from N = 5 mice.

**Table 2:** Animal-level two-way mixed-design repeated-measures ANOVA of EMG event parameters in young and aged mice with and without PV-IN photostimulation.

Parameter	Age, F(1,10)	Age, *p*	+PV, F(1,10)	PV Stim., *p*	Age × PV, F(1,10)	Interaction, *p*	Young, Šídák *p*	Aged, Šídák *p*
**Amplitude**	2.2153	0.1675	8.2702	0.0165	8.0852	0.0174	0.9977	0.0621
**Duration**	1.9620	0.1916	2.8019	0.1251	2.1733	0.1712	0.2259	0.9806
**IEI**	8.2218	0.0167	0.0123	0.9137	2.0408	0.1836	0.7303	0.3366
**Frequency**	15.4314	0.0028	0.0156	0.9030	6.0769	0.0334	0.1081	0.3632

Exact F and *p*-values are shown. For all EMG analyses, N = 6 mice per age group. Detected events (34 from young mice and 19 from aged mice) were averaged within each animal and condition before statistical analysis and were not treated as independent biological replicates. Šídák-adjusted *p*-values are reported for within-age comparisons of control versus PV-IN photostimulation.

## Data Availability

The original contributions presented in this study are included in the article. Further inquiries can be directed to the corresponding author(s).
